# Differences in clinical features and dengue severity between local and migrant Chinese with dengue infection in Singapore

**DOI:** 10.1371/journal.pone.0201441

**Published:** 2018-08-15

**Authors:** Chuanhui Xu, Junxiong Pang, Jung Pu Hsu, Yee Sin Leo, David Chien Boon Lye

**Affiliations:** 1 Communicable Disease Centre, Institute of Infectious Diseases and Epidemiology, Tan Tock Seng Hospital, Singapore; 2 Saw Swee Hock School of Public Health, National University of Singapore, Singapore; 3 Yong Loo Lin School of Medicine, National University of Singapore, Singapore; 4 Lee Kong Chian School of Medicine, Nanyang Technological University, Singapore; CEA, FRANCE

## Abstract

Dengue is endemic in Singapore but not China. We compared clinical features and disease severity of dengue between local and migrant Chinese, most of whom were construction workers, in Singapore. A retrospective study with all hospitalized dengue patients from 2005 to 2008 were performed, including 2609 local and 1195 migrant Chinese. Compared with local Chinese, migrant Chinese were younger. There were more males, but fewer had comorbidities. Migrant Chinese had more headache, eye pain, nausea and myalgia. They had significantly lower median leukocyte count, ALT and AST, and higher platelet count nadir. Among warning signs, migrant Chinese had significantly less persistent vomiting, clinical fluid accumulation, hepatomegaly, hematocrit rise with rapid platelet drop, and more mucosal bleeding. Adjusted for age, gender and comorbidities, migrant Chinese were significantly at higher risk of dengue hemorrhagic fever (DHF) (adjusted odds ratio [aOR]: 1.20, 95% confidence interval [CI]: 1.03–1.41) and dengue shock syndrome (aOR: 1.49, 95% CI: 1.06–2.10), and had longer hospitalization (β coefficient value: 0.27, 95%CI: 0.09–0.44, *p* = 0.003). There was 1 death among migrant Chinese and 2 deaths among local Chinese. We documented differences in clinical and laboratory features, and dengue severity between local and migrant Chinese in Singapore. Migrant Chinese may need more medical attention given higher risk of DHF.

## Introduction

Dengue is endemic in tropical and subtropical regions with rapid spread internationally [1]. The global disease burden is estimated to be as high as 390 million infections annually [2]. Moreover, the incidence of dengue cases has increased 30 fold with expanding geographic distribution to new countries in last 5 decades [3]. The spectrum of clinical manifestations of dengue is protean, from asymptomatic to mildly symptomatic, to life-threatening disease resulting in death [4].

There are a few risk factors for severe dengue identified by epidemiologic studies. Young children have an increased risk of severe dengue [5, 6]. There is an association between female gender and dengue shock syndrome (DSS) [5]. In addition, secondary dengue infection [7] or dengue virus serotype 2 (DENV-2) [8] are associated with severe dengue.

Disease manifestations and dengue severity might be influenced by genetic background. In Cuba, DSS affected more white population compared with non-white populations [9, 10]. A variant in CD209 promoter was identified to be associated with dengue severity [11]. In addition, a genome-wide association study among Vietnamese patients showed genetic variants of the human major-histocompatibility-complex class I polypeptide-related sequence B and phospholipase C epsilon 1 genes as risk factors for dengue shock syndrome [12]. However, only a few studies were conducted to investigate the differences of clinical features of dengue among different ethnicities and migrants. Studies from Latin America showed that Afro-Colombian was a protective factor against dengue fever [13], and Afro-Brazilian ethnicity and African ancestry a protective factor against DHF [14]. Migrant workers in Thailand, mainly Burmese and Cambodians, had a lower risk of dengue compared with local Thai workers [15].

Singapore is ethnically diverse with the general population comprising 74.3% Chinese, 13.4% Malays, 9.1% Indians and 3.2% others [16]. In addition, Singapore has a significant proportion of migrant worker populations, with about 1.4 million migrant workers out of 5.6 million total population [17]. An 8-year, multicenter longitudinal prospective study in Singapore found that Malay ethnicity was a protective factor and foreign workers in dormitories a risk factor for dengue [18]. A study in Singapore found dengue infection among migrant Chinese workers was characterized by prominent gastrointestinal symptoms [19]. In our previous study on adult dengue in Singapore, we observed that the majority of dengue patients were Chinese (74.2%), with Malays and Indians accounting for 5.3% and 9.2% respectively [20]. Overall, 56% of our study cohort was Singapore permanent residents and citizens. Given the differences of socioeconomic status, prior dengue exposure, and similar genetic background between local Singaporean Chinese and migrant Chinese workers from China, we aimed to compare their clinical manifestations and disease severity.

## Materials and methods

### Study population and data collection

A retrospective observational study was performed in the Department of Infectious Diseases at Tan Tock Seng Hospital, Singapore, which is a governmental hospital and tertiary referral centre for dengue patients. The study population comprised all adult dengue patients admitted from 1 January 2005 to 31 December 2008. Probable dengue diagnosis required acute fever and two or more of headache, eye pain, myalgia, arthralgia, rash, hemorrhagic manifestations or leukopenia [21], or acute fever with two or more of nausea or vomiting, rash, aches and pains, leukopenia or any warning signs [3]. Dengue fever patients had positive acute dengue serology, as measured by Dengue Duo IgM & IgG Rapid Strip Test (Panbio Diagnostic, Queensland, Australia) [22, 23], and fulfilled diagnostic criteria of probable dengue based on World Health Organization (WHO) 1997 [21] or 2009 dengue guidelines [3]. Confirmed dengue patients had positive dengue polymerase chain reaction (PCR) assay [24]. Patients were eligible for analysis if they were of Chinese ethnicity, had data on country of origin, and fulfilled the criteria of laboratory confirmation with compatible clinical syndrome. Local Singaporean Chinese referred to patients with Singaporean citizenship with Chinese ethnicity, and migrant Chinese referred to patients from China with Chinese citizenship.

Data were collected by medically trained research assistants from hospital medical records including a standardized dengue care path during the study period. Data collected for all patients included age, gender, ethnicity, nationality, comorbidities, Charlson’s comorbidity score, clinical signs and symptoms, laboratory and radiological investigations, and clinical outcome data including dengue severity, intensive care unit (ICU) admission, hospital length of stay and death. Among laboratory data, nadir level of white blood cells, lymphocytes, neutrophils, platelet, protein and albumin, and maximum level of hematocrit, creatinine, bilirubin, ALT and AST were extracted during hospitalization. Consistent data collection was guided by pre-defined data specification guideline, automatic checks within Microsoft Access, data verification check and 10% random double data entry.

Dengue severity was classified into DHF and DSS based on WHO 1997 dengue guideline [21], and dengue with warning signs and severe dengue based on WHO 2009 dengue guideline [3]. The classification was based on data collected from hospital admission to discharge. A diagnosis of DHF required all four of fever, thrombocytopenia <100x10^9^/L, hemorrhagic manifestations and plasma leakage, while a diagnosis of DSS required the presence of DHF with either tachycardia with narrow pulse pressure <20mmHg or systolic hypotension for age [21]. Warning signs of dengue included: persistent vomiting (vomiting during two or more consecutive days), abdominal pain or tenderness, mucosal bleeding, clinical fluid accumulation, lethargy or restlessness, hepatomegaly or serum hematocrit rise with rapid platelet count drop (hematocrit change > = 20% concurrent with platelet < 50,000/ μL) [3, 25]. Severe dengue comprised severe plasma leakage, severe bleeding or severe organ impairment [3]. Severe bleeding was defined as gastrointestinal bleeding, requirement of packed red cell or whole blood transfusion, or menorrhagia. Severe plasma leakage was defined as hematocrit change > = 20%, pleural effusion or ascites associated with respiratory distress or shock. Severe organ impairment was defined as liver (AST or ALT > = 1000) or acute renal impairment (creatinine > = 2 times upper limit normal for age and sex), encephalopathy or encephalitis or myocarditis [25].

### Statistical methods

The descriptive results for categorical variables were displayed by frequency and percentage. For continuous variables, median and ranges were used. To compare differences of clinical and laboratory parameters, severity and outcomes of dengue between local and migrant Chinese, Chi-square tests were performed for categorical variables, and Mann-Whitney *u* test for non-parametric continuous variables. With adjustment for patients’ confounding variables, i.e. age, gender, nationality and comorbidities, logistic multivariate regression for categorical variables and linear multivariate regression for continuous variables were performed respectively. Data analyses were done using STATA software version 13 (Stata Corp., College Station, TX). All tests were conducted at the 5% level of significance, with odd ratio (OR), β coefficient value, *p*-value and corresponding 95% confidence interval (CI) reported where applicable.

### Ethics approval

This study was approved by Domain Specific Review Board, National Healthcare Group, Singapore (DSRB-E/08/567) with waiver of informed consent as this was a retrospective study and the data were analyzed anonymously. All methods were performed in accordance with the relevant guidelines and regulations.

## Results

### Clinical features among local and migrant Chinese patients with dengue

Of 3804 patients with dengue and Chinese ethnicity, 2609 were local Singaporean Chinese and 1195 migrant Chinese. For demographic data (Table 1), migrant Chinese were younger compared to local Chinese (35 vs. 39 years old, *p*<0.001). Among migrant Chinese, there were more male patients (78%) compared with local Chinese (58%) (*p*<0.001). Notably, there were significantly fewer comorbidities among migrant Chinese compared with local Chinese, i.e. hypertension (2% vs. 13%, *p*<0.001), diabetes mellitus (0.3% vs. 4.5%, *p*<0.001). Charlson’s comorbidity score was also significantly lower among migrant Chinese (*p*<0.001).

**Table 1:** Characteristics of clinical and laboratory features among local and migrant adult dengue Chinese patients in Singapore.

Variables	Local (n = 2609)	Migrant (n = 1195)		*P* value
**Demographic**				
Age	39(25–49)	35(28–39)		<0.001
Male gender (%)	1507 (58)	930 (78)		<0.001
**Co-morbidities**				
Hypertension	340 (13)	18 (2)		<0.001
Diabetes mellitus	117 (4.5)	4 (0.3)		<0.001
Charlson’s comorbidity score	0 (0–0)	0 (0–0)		<0.001
**Clinical features**				
Fever	2601 (99.7)	1193 (99.8)		0.436
Duration of fever on admission (days)	5(4–6)	5(4–6)		<0.001
Headache	1216 (46.6)	681 (57.0)		<0.001
Eye pain	59 (2.3)	46 (3.8)		0.006
Nausea	1593 (61.1)	829 (69.4)		<0.001
Vomiting	1142 (43.8)	533 (44.6)		0.632
Diarrhea	1001 (38.4)	439 (36.7)		0.336
Myalgia	1789 (68.6)	871 (72.9)		0.007
Arthralgia	293 (11.2)	156 (13.1)		0.106
Rash	2192 (84.0)	1018 (85.2)		0.356
Clinical bleeding	1853 (71.0)	819 (68.5)		0.119
Leukopenia	2081 (79.8)	1107 (92.6)		<0.001
**Laboratory investigations**				
WBC (X 10^9^/L)	2.4(1.8–3.3)	1.9(1.4–2.5)		<0.001
Lymphocyte (%)	23.7(17–31.75)	24(18–32)		0.147
Neutrophil (%)	30.9(23.1–38.2)	33.2(26.5–40.1)		<0.001
HCT (%)	44.8(41.6–47.8)	45.1(42.4–47.3)		0.411
Platelet nadir (X 10^9^/L)	40(20–63)	45(28–61)		<0.001
Creatinine (umol/L)	76(62–90)	74(65–85)		0.078
Protein (g/L)	66(61–70)	65(60–69)		<0.001
Albumin (g/L)	37(34–40)	38(35–41)		<0.001
Bilirubin (umol/L)	11(8–15)	12(9–15)		0.124
ALT (U/L)	82(45–165)	52(29–108)		<0.001
AST (U/L)	130(76–243)	87(52–171)		<0.001

Median (interquartile range) for non-parametric continuous variables, n (%) for categorical variables

Chi-square test for categorical variables, and Mann-Whitney u test for non-parametric continuous variables

Of clinical features and laboratory parameters from hospital admission to discharge, there were many significant differences between local and migrant Chinese (Table 1). Compared with local Chinese, migrant Chinese had more headache (57.0% vs. 46.6%, *p*<0.001), eye pain (3.8% vs. 2.3%, *p* = 0.006), nausea (69.4% vs. 61.1%, *p*<0.001) and myalgia (72.9% vs. 68.6%, *p* = 0.007). They had significantly lower median leukocyte count (1.9 vs. 2.4 x10^9^/L, *p*<0.001), ALT (52 vs. 82 U/L) and AST (87 vs. 130U/L), and higher platelet count nadir (45 vs. 40 x10^9^/L, *p*<0.001).

### Severity and outcomes among different population

We analyzed the differences of disease severity and outcomes between local and migrant Chinese patients with dengue (Table 2). In terms of warning signs, significantly less persistent vomiting (4.0% vs. 6.4%, *p*<0.001), clinical fluid accumulation (1.4% vs. 5.3%, *p*<0.001), hepatomegaly (1.8% vs. 3.8%, *p* = 0.001), and hematocrit rise with rapid platelet drop (9.5% vs. 14.6%, *p*<0.001) were found among migrant versus local Chinese. Notably, there were more mucosal bleeding (45.5% vs. 30.9%, *p*<0.001) among migrant versus local Chinese. There was no difference with regard to abdominal pain/tenderness and lethargy. Overall the presence of any warning sign was significantly higher among migrant (77.4%) than local Chinese (73.9%) (*p* = 0.02). However, local Chinese were more likely to have severe dengue than migrant Chinese (17.7% vs. 12.6%, *p* < 0.001). In subgroup analysis, there were more patients with severe plasma leakage (8.3% vs. 4.6%, *p* < 0.001) and severe organ impairment (3.3% vs. 1.0%, *p* = 0.013) among local versus migrant Chinese. There was no difference in terms of DHF, DSS, length of hospitalization or ICU admission, death between these two populations. The findings of disease severity and outcomes were similar at presentation among local and migrant adult dengue Chinese patients in Singapore (S1 Table).

**Table 2:** Severity and outcomes among local and migrant adult dengue Chinese patients in Singapore.

	Local (2609)	Migrant (1195)	*P* value
**WHO 2009 warning signs**			
Any warning signs	1929 (73.9)	925 (77.4)	0.022
Abdominal pain	964 (36.9)	418 (35.0)	0.241
Persistent vomiting	168 (6.4)	48 (4.0)	<0.001
Clinical fluid accumulation	138 (5.3)	17 (1.4)	<0.001
Mucosal bleeding	806 (30.9)	544 (45.5)	<0.001
Hepatomegaly	98 (3.8)	21 (1.8)	0.001
lethargy	899 (34.5)	420 (35.1)	0.679
Hematocrit rise with rapid platelet drop	381 (14.6)	114 (9.5)	<0.001
DHF	749 (28.7)	349 (29.2)	0.754
DSS	111 (4.3)	60 (5.0)	0.290
Severe dengue	461 (17.7)	151(12.6)	<0.001
Severe plasma leakage	217 (8.3)	55 (4.6)	<0.001
Severe bleeding	233 (8.9)	86 (7.2)	0.073
Severe organ impairment	86 (3.3)	13 (1.0)	0.013
LOS hospitalization (days)	4(3–5)	4(3–5)	0.120
ICU admission	8 (0.3)	2 (0.2)	0.436
Death	2 (0.08)	1 (0.08)	1.000

Median (interquartile range) for non-parametric continuous variables, n (%) for categorical variables

Chi-square test for categorical variables, and Mann-Whitney *u* test for non-parametric continuous variables

DHF: dengue hemorrhagic fever; DSS: dengue shock syndrome; ICU: intensive care unit; LOS: length of stay.

Given the differences of in baseline characteristics of these two populations, multivariate regression analyses were performed with adjustment for age, gender, nationality and comorbidities (Table 3 and S2 Table). Migrant Chinese were significantly at higher risk of DHF (adjusted odds ratio [aOR]: 1.20, 95% confidence interval [CI]: 1.03–1.41, *p* = 0.024) and DSS (aOR: 1.49, 95%CI: 1.06–2.10, *p* = 0.021), and lower risk of severe plasma leakage (aOR: 0.66, 95%CI: 0.48–0.90, *p* = 0.009) and severe organ impairment (aOR: 0.45, 95%CI: 0.25–0.84, *p* = 0.011), compared with local Chinese. Migrant Chinese had significantly longer hospitalization (β coefficient value: 0.27, 95%CI: 0.09–0.44, *p* = 0.003).

**Table 3:** Migrant status as a risk factor for disease severity and outcomes.

	Local	Migrant	
		AOR/β (95% CI)	*p* value
DHF	Referent	1.20(1.03–1.41)	0.024
DSS		1.49(1.06–2.10)	0.021
Severe dengue		0.88(0.72–1.09)	0.242
Severe plasma leakage		0.66 (0.48–0.90)	0.009
Severe bleeding		1.10(0.83–1.44)	0.512
Severe organ involvement		0.45 (0.25–0.84)	0.011
ICU admission		0.92 (0.17–4.91)	0.919
LOS of hospitalization*		0.27 (0.09–0.44)	0.003

Logistic multivariate regression for categorical variables and linear multivariate regression for continuous variables, respectively.

Migrant as a risk factor was adjusted to patient’s background variables, i.e. age, gender, and co-morbidities.

* linear multivariate regression performed, and the β coefficient value was shown.

AOR: adjusted odd ratio. DHF: dengue hemorrhagic fever; DSS: dengue shock syndrome; ICU: intensive care unit; LOS: length of stay.

To understand the underlying cause of differences of disease severity between local and migrant Chinese with dengue infection, we examined the environmental factors given their similar genetic background. In Singapore, dengue infections were predominantly due to dengue serotype 1 (detected in 75% to 100% of dengue samples collected each month) during the epidemic in the years 2005–2006, and dengue serotype 2 (detected in up to 91% of dengue samples) during the epidemic in the years 2007–2008 [26]. Hence we analyzed the disease severity in the years 2005–2006 and 2007–2008, respectively. As shown in Table 4, in the years 2005–2006, migrant Chinese were significantly at higher risk of DSS (aOR: 1.58, 95%CI: 1.02–2.45, p = 0.041), and lower risk of severe plasma leakage (aOR: 0.72, 95%CI: 0.57–0.92, p = 0.009) and severe organ impairment (aOR: 0.47, 95%CI: 0.24–0.92, p = 0.027), compared with local Chinese. The migrant Chinese had significantly longer hospitalization (β coefficient value: 0.43, 95%CI: 0.20–0.66, p < 0.001). However, such differences were not found in the years 2007–2008.

**Table 4:** Migrant status as a risk factor for disease severity and outcomes in the year 2005–2006 and year 2007–2008.

	2005–2006			2007–2008		
	Local (1927)	Migrant (812)		local (682)	Migrant (383)	
		aOR/β (95% CI)	p value		aOR/β (95% CI)	p value
DHF	Referent	1.12(0.92–1.37)	0.262	Referent	1.07(0.81–1.42)	0.617
DSS		1.58(1.02–2.45)	0.041		1.03(0.58–1.80)	0.928
Severe dengue		0.79(0.61–1.02)	0.074		0.94(0.64–1.38)	0.752
Severe plasma leakage		0.72 (0.57–0.92)	0.009		0.52 (0.35–0.78)	0.002
Severe bleeding		0.83(0.57–1.19)	0.314		1.24(0.78–1.98)	0.362
Severe organ involvement		0.47 (0.24–0.92)	0.027		0.47 (0.09–2.30)	0.349
ICU admission		0.94 (0.18–5.09)	0.95		NA	NA
LOS of hospitalization*		0.43 (0.20–0.66)	< 0.001		0.04 (-0.17–0.25)	0.708

Logistic multivariate regression for categorical variables and linear multivariate regression for continuous variables, respectively.

Migrant as a risk factor was adjusted to patient’s background variables, i.e. age, gender, and co-morbidities.

* linear multivariate regression performed, and the β coefficient value was shown.

aOR: adjusted odd ratio. DHF: dengue hemorrhagic fever; DSS: dengue shock syndrome; ICU: intensive care unit; LOS: length of stay.

## Discussion

In this study, migrant Chinese were younger and predominantly male, and had fewer comorbidities, which is consistent with the fact that they were younger migrant workers. In terms of clinical characteristics, migrant Chinese were more symptomatic, among which there was remarkably more headache. There was an outbreak of dengue among migrant Chinese workers with more gastroenterological symptoms [19]; however, this was not found in our study. This discrepancy might be explained by the small cohort (n = 37) in the previous study which may not be representative of the whole migrant Chinese population. Moreover, dengue epidemics had spread from southern coastal regions to relatively northern and central regions where most migrant Chinese workers come from [27]. This might result in a different immunologic status of these migrant workers which may modify disease manifestations and severity.

Notably, there was significantly more mucosal bleeding among migrant Chinese. Our previous study showed the presence of bleeding (OR 237.6), hypoproteinemia (OR 1.28) and lymphopenia (OR 1.08) could be simple clinical and laboratory variables to predict DHF [28]. Migrant Chinese had significantly more mucosal bleeding and lower serum protein in our study. After adjustment for age, gender and comorbidities, migrant Chinese were at higher risk of DHF. Not surprisingly, age, female gender and diabetes mellitus were again found as a risk factor for DHF (S2 Table), in keeping with our previous study [29], as well as studies in Taiwan [30] and Cuba [31]. Our study is the first report that showed the migrant status of the same ethnicity is an independent risk factor for DHF and DSS, and longer hospitalization after adjustment for age, gender, and comorbidities. Three plausible reasons can be postulated. Firstly, serological status might play an important role in the differences of disease severity and outcomes. As observed in our study, when the dengue outbreak was predominantly dengue serotype 2 in the years 2007–2008, there were no differences among local and migrant Chinese. However, the differences were still found in the years 2005–2006 when dengue outbreak was predominantly serotype 1. As most migrant Chinese are construction workers and live in crowded dormitory, they might be more prone to contract dengue serotype 2. A serological survey done among migrant Chinese construction workers showed the dengue virus were DENV-2 [19]. Secondly, most of the migrant Chinese workers were from rural area whereas local Chinese were from urban Singapore, which may influence their nutritional and immunological status. Thirdly, the migrant Chinese workers presented to hospital later in their course of illness possibly resulting in more severe disease.

There were a few limitations to our retrospective study. Firstly, there may be a selection bias because this was a hospital-based study. Most dengue patients are asymptomatic or mildly symptomatic, and may not seek medical care, which might be more common among migrant Chinese workers. Hence, the hospitalized migrant Chinese could have more severe disease because of this selection bias. Secondly, there might be undetected comorbidities among migrant Chinese migrants, such as diabetes mellitus and hypertension because of limited access to health care. Thirdly, it would be ideal to have the serologic data of prior dengue infection which is a known risk factor of DHF [22, 32]. Unfortunately, IgG result was only available for 33% of our patients.

To our knowledge, this is the first study to document the different clinical manifestations of the same ethnicity with different social backgrounds. The differences in clinical characteristics and disease severity may indicate the impact of serological status, immunologic background, comorbidities and socioeconomic status on dengue. The migrant Chinese in Singapore with dengue warrant more medical attention, given greater risk of DHF.

## Supporting information

S1 Table Severity and outcomes at presentation among local and migrant adult dengue Chinese patients in Singapore. (DOCX)

S2 Table Multivariate regression analyses for disease severity and outcomes. (DOCX)

## References

[1] Guzman MG, Halstead SB, Artsob H, Buchy P, Farrar J, Gubler DJ, et al Dengue: a continuing global threat. Nat Rev Microbiol. 2010;8(12 Suppl):S7–16. 10.1038/nrmicro2460 ; PubMed Central PMCID: PMCPMC4333201. 21079655 PMC4333201

[2] Bhatt S, Gething PW, Brady OJ, Messina JP, Farlow AW, Moyes CL, et al The global distribution and burden of dengue. Nature. 2013;496(7446):504–7. 10.1038/nature12060 ; PubMed Central PMCID: PMCPMC3651993. 23563266 PMC3651993

[3] Dengue guidelines for diagnosis, treatment, prevention and control. 3rd edition. World Health Organisation. http://www.who.int/tdr/publications/documents/dengue-diagnosis.pdf. Accessed 27 May 2017. 23762963

[4] Simmons CP, Farrar JJ, Nguyen v V, Dengue WB. N Engl J Med. 2012;366(15):1423–32. 22494122 10.1056/NEJMra1110265

[5] Anders KL, Nguyet NM, Chau NV, Hung NT, Thuy TT, Lien le B, et al Epidemiological factors associated with dengue shock syndrome and mortality in hospitalized dengue patients in Ho Chi Minh City, Vietnam. Am J Trop Med Hyg. 2011;84(1):127–34. 10.4269/ajtmh.2011.10-0476 ; PubMed Central PMCID: PMCPMC3005500. 21212214 PMC3005500

[6] Pham TB, Nguyen TH, Vu TQ, Nguyen TL, Malvy D. [Predictive factors of dengue shock syndrome at the children Hospital No. 1, Ho-chi-Minh City, Vietnam]. Bull Soc Pathol Exot. 2007;100(1):43–7. 1742695. 17402695

[7] Sangkawibha N, Rojanasuphot S, Ahandrik S, Viriyapongse S, Jatanasen S, Salitul V, et al Risk factors in dengue shock syndrome: a prospective epidemiologic study in Rayong, Thailand. I. The 1980 outbreak. Am J Epidemiol. 1984;120(5):653–69. . 6496446 10.1093/oxfordjournals.aje.a113932

[8] Pichainarong N, Mongkalangoon N, Kalayanarooj S, Chaveepojnkamjorn W. Relationship between body size and severity of dengue hemorrhagic fever among children aged 0–14 years. Southeast Asian J Trop Med Public Health. 2006;37(2):283–8. . 17124987

[9] Gonzalez D, Castro OE, Kouri G, Perez J, Martinez E, Vazquez S, et al Classical dengue hemorrhagic fever resulting from two dengue infections spaced 20 years or more apart: Havana, Dengue 3 epidemic, 2001–2002. Int J Infect Dis. 2005;9(5):280–5. 10.1016/j.ijid.2004.07.012 . 16023878

[10] Guzman MG, Alvarez M, Rodriguez R, Rosario D, Vazquez S, Vald s L, et al Fatal dengue hemorrhagic fever in Cuba, 1997. Int J Infect Dis. 1999;3(3):130–5. . 10460923 10.1016/s1201-9712(99)90033-4

[11] Sakuntabhai A, Turbpaiboon C, Casademont I, Chuansumrit A, Lowhnoo T, Kajaste-Rudnitski A, et al A variant in the CD209 promoter is associated with severity of dengue disease. Nat Genet. 2005;37(5):507–13. 10.1038/ng1550 . 15838506 PMC7096904

[12] Khor CC, Chau TN, Pang J, Davila S, Long HT, Ong RT, et al Genome-wide association study identifies susceptibility loci for dengue shock syndrome at MICB and PLCE1. Nat Genet. 2011;43(11):1139–41. 10.1038/ng.960 ; PubMed Central PMCID: PMCPMC3223402. 22001756 PMC3223402

[13] Rojas Palacios JH, Alzate A, Martinez Romero HJ, Concha-Eastman AI. AfroColombian ethnicity, a paradoxical protective factor against Dengue. Colomb Med (Cali). 2016;47(3):133–41. ; PubMed Central PMCID: PMCPMC5091271. 27821892 PMC5091271

[14] Blanton RE, Silva LK, Morato VG, Parrado AR, Dias JP, Melo PR, et al Genetic ancestry and income are associated with dengue hemorrhagic fever in a highly admixed population. Eur J Hum Genet. 2008;16(6):762–5. 10.1038/ejhg.2008.4 . 18270538

[15] Rakprasit J, Nakamura K, Seino K, Morita A. Healthcare use for communicable diseases among migrant workers in comparison with Thai workers. Ind Health. 2017;55(1):67–75. 10.2486/indhealth.2016-0107 ; PubMed Central PMCID: PMCPMC5285315. 27568679 PMC5285315

[16] Department of statistics Singapore. http://www.singstat.gov.sg/docs/default-source/default-document-library/publications/publications_and_papers/population_and_population_structure/population2016.pdf. Accessed 27 May 2017

[17] Ministry of Manpower, Singapore. Foreign Workforce Numbers. http://www.mom.gov.sg/documents-and-publications/foreign-workforce-numbers. Accessed 27 May 2017.

[18] Yung CF, Chan SP, Thein TL, Chai SC, Leo YS. Epidemiological risk factors for adult dengue in Singapore: an 8-year nested test negative case control study. BMC Infect Dis. 2016;16:323 10.1186/s12879-016-1662-4 ; PubMed Central PMCID: PMCPMC4938976. 27390842 PMC4938976

[19] Seet RC, Ooi EE, Wong HB, Paton NI. An outbreak of primary dengue infection among migrant Chinese workers in Singapore characterized by prominent gastrointestinal symptoms and a high proportion of symptomatic cases. J Clin Virol. 2005;33(4):336–40. 10.1016/j.jcv.2005.03.002 . 16036184

[20] Gan VC, Lye DC, Thein TL, Dimatatac F, Tan AS, Leo YS. Implications of discordance in world health organization 1997 and 2009 dengue classifications in adult dengue. PLoS One. 2013;8(4):e60946 10.1371/journal.pone.0060946 ; PubMed Central PMCID: PMCPMC3613419. 23573291 PMC3613419

[21] Dengue guidelines for diagnosis, treatment, prevention and control. 2nd edition. World Health Organisation. http://whqlibdoc.who.int/publications/1997/9241545003_eng.pdf. Accessed 27 May 2017. 23762963

[22] Cuzzubbo AJ, Endy TP, Nisalak A, Kalayanarooj S, Vaughn DW, Ogata SA, et al Use of recombinant envelope proteins for serological diagnosis of Dengue virus infection in an immunochromatographic assay. Clin Diagn Lab Immunol. 2001;8(6):1150–5. 10.1128/CDLI.8.6.1150-1155.2001 ; PubMed Central PMCID: PMCPMC96242. 11687456 PMC96242

[23] Blacksell SD, Newton PN, Bell D, Kelley J, Mammen MP Jr., Vaughn DW, et al The comparative accuracy of 8 commercial rapid immunochromatographic assays for the diagnosis of acute dengue virus infection. Clin Infect Dis. 2006;42(8):1127–34. 10.1086/501358 . 16575730

[24] Barkham TM, Chung YK, Tang KF, Ooi EE. The performance of RT-PCR compared with a rapid serological assay for acute dengue fever in a diagnostic laboratory. Trans R Soc Trop Med Hyg. 2006;100(2):142–8. 10.1016/j.trstmh.2005.05.015 . 16212996 PMC7107224

[25] Leo YS, Thein TL, Fisher DA, Low JG, Oh HM, Narayanan RL, et al Confirmed adult dengue deaths in Singapore: 5-year multi-center retrospective study. BMC Infect Dis. 2011;11:123 10.1186/1471-2334-11-123 ; PubMed Central PMCID: PMCPMC3112097. 21569427 PMC3112097

[26] Lee KS, Lai YL, Lo S, Barkham T, Aw P, Ooi PL, et al Dengue virus surveillance for early warning, Singapore. Emerg Infect Dis. 2010;16(5):847–9. 10.3201/eid1605.091006 ; PubMed Central PMCID: PMCPMC2953985. 20409381 PMC2953985

[27] Wu JY, Lun ZR, James AA, Chen XG. Dengue Fever in mainland China. Am J Trop Med Hyg. 2010;83(3):664–71. 10.4269/ajtmh.2010.09-0755 ; PubMed Central PMCID: PMCPMC2929067. 20810836 PMC2929067

[28] Lee VJ, Lye DC, Sun Y, Fernandez G, Ong A, Leo YS. Predictive value of simple clinical and laboratory variables for dengue hemorrhagic fever in adults. J Clin Virol. 2008;42(1):34–9. 10.1016/j.jcv.2007.12.017 . 18282738

[29] Pang J, Salim A, Lee VJ, Hibberd ML, Chia KS, Leo YS, et al Diabetes with hypertension as risk factors for adult dengue hemorrhagic fever in a predominantly dengue serotype 2 epidemic: a case control study. PLoS Negl Trop Dis. 2012;6(5):e1641 10.1371/journal.pntd.0001641 ; PubMed Central PMCID: PMCPMC3341340. 22563519 PMC3341340

[30] Lee MS, Hwang KP, Chen TC, Lu PL, Chen TP. Clinical characteristics of dengue and dengue hemorrhagic fever in a medical center of southern Taiwan during the 2002 epidemic. J Microbiol Immunol Infect. 2006;39(2):121–9. . 16604244

[31] Guzman MG, Kouri GP, Bravo J, Soler M, Vazquez S, Santos M, et al Dengue haemorrhagic fever in Cuba. II. Clinical investigations. Trans R Soc Trop Med Hyg. 1984;78(2):239–41. . 6464114 10.1016/0035-9203(84)90286-4

[32] Thomas L, Verlaeten O, Cabie A, Kaidomar S, Moravie V, Martial J, et al Influence of the dengue serotype, previous dengue infection, and plasma viral load on clinical presentation and outcome during a dengue-2 and dengue-4 co-epidemic. Am J Trop Med Hyg. 2008;78(6):990–8. . 18541782

