# Supplementary material for: Differences in clinical features and dengue severity between local and migrant Chinese with dengue infection in Singapore
Source: PLoS One. 2018 Aug 15;13(8):e0201441. doi: 10.1371/journal.pone.0201441 (PMC6093606; doi:10.1371/journal.pone.0201441)
Supplement: S1 Table — (DOCX) [file pone.0201441.s001.docx]

S1 Table: Severity and outcomes at presentation among local and migrant adult dengue Chinese patients in Singapore.

|  | Local (2609) | Migrant (1195) | *P* value |
| --- | --- | --- | --- |
| DHF | 466 (17.9) | 223 (18.7) | 0.553 |
| DSS | 31 (1.2) | 18 (1.5) | 0.419 |
| Severe dengue | 316 (12.1) | 89 (7.4) | <0.001 |
| Severe plasma leakage | 127 (4.9) | 28 (2.3) | <0.001 |
| Severe bleeding | 155 (5.9) | 57 (4.8) | 0.144 |
| Severe organ impairment | 62 (2.4) | 6 (0.5) | <0.001 |
| ICU admission | 8 (0.3) | 2 (0.2) | 0.436 |

Median (interquartile range) for non-parametric continuous variables, n (%) for categorical variables

Chi-square test for categorical variables, and Mann-Whitney *u* test for non-parametric continuous variables

DHF: dengue hemorrhagic fever; DSS: dengue shock syndrome; ICU: intensive care unit; LOS: length of stay.
