# Supplementary material for: Differences in clinical features and dengue severity between local and migrant Chinese with dengue infection in Singapore
Source: PLoS One. 2018 Aug 15;13(8):e0201441. doi: 10.1371/journal.pone.0201441 (PMC6093606; doi:10.1371/journal.pone.0201441)
Supplement: S2 Table — (DOCX) [file pone.0201441.s002.docx]

S2 Table: Multivariate regression analyses for disease severity and outcomes.

|  | |  |  |  | | |  |  |
| --- | --- | --- | --- | --- | --- | --- | --- | --- |
| DHF | | Odds Ratio | P | 95% CI | | | |  |
| Age | | 1.02 | 0.000 | 1.01 | | 1.02 | |  |
| Male | | 0.66 | 0.000 | 0.57 | | 0.77 | |  |
| Migrant | | 1.20 | 0.024 | 1.02 | | 1.41 | |  |
| Diabetes mellitus | | 1.56 | 0.042 | 1.02 | | 2.41 | |  |
| Hypertension | | 0.74 | 0.036 | 0.56 | | 0.98 | |  |
| Charlson’s comorbidity score | | 1.10 | 0.232 | 0.94 | | 1.28 | |  |
|  | |  |  | |  | | |  |
| DSS | | Odds Ratio | P | 95% CI | | | |  |
| Age | | 1.00 | 0.449 | 0.98 | | 1.01 | |  |
| Male | | 0.29 | 0.000 | 0.21 | | 0.40 | |  |
| Migrant | | 1.49 | 0.021 | 1.06 | | 2.10 | |  |
| Diabetes mellitus | | 0.85 | 0.770 | 0.30 | | 2.45 | |  |
| Hypertension | | 0.49 | 0.067 | 0.23 | | 1.05 | |  |
| Charlson’s comorbidity score | | 1.34 | 0.013 | 1.06 | | 1.70 | |  |
|  | |  |  | |  | | |  |
| Severe Dengue | | Odds Ratio | P | 95% CI | | | |  |
| Age | | 1.00 | 0.318 | 0.99 | | 1.00 | |  |
| Male | | 0.35 | 0.000 | 0.29 | | 0.42 | |  |
| Migrant | | 0.88 | 0.242 | 0.72 | | 1.09 | |  |
| Diabetes mellitus | | 1.44 | 0.143 | 0.88 | | 2.35 | |  |
| Hypertension | | 1.17 | 0.349 | 0.84 | | 1.62 | |  |
| Charlson’s comorbidity score | | 1.21 | 0.022 | 1.03 | | 1.44 | |  |
|  | |  |  | |  | |  |  |
| Severe Bleeding | | Odds Ratio | P | 95% CI | | | |  |
| Age | | 1.00 | 0.391 | 0.99 | | 1.01 | |  |
| Male | | 0.22 | 0.000 | 0.17 | | 0.28 | |  |
| Migrant | | 1.10 | 0.512 | 0.83 | | 1.44 | |  |
| Diabetes mellitus | | 0.82 | 0.580 | 0.40 | | 1.67 | |  |
| Hypertension | | 0.94 | 0.793 | 0.60 | | 1.48 | |  |
| Charlson’s comorbidity score | | 1.26 | 0.026 | 1.03 | | 1.53 | |  |
|  | |  |  | |  | |  |  |
| Severe Organ impairment | Odds Ratio | | P | 95% CI | | | |  |
| Age | 1.00 | | 0.856 | 0.99 | | 1.02 | |  |
| Male | 0.63 | | 0.030 | 0.41 | | 0.96 | |  |
| Migrant | 0.45 | | 0.011 | 0.25 | | 0.84 | |  |
| Diabetes mellitus | 5.48 | | 0.000 | 2.26 | | 13.27 | |  |
| Hypertension | 1.25 | | 0.506 | 0.64 | | 2.44 | |  |
| Charlson’s comorbidity score | 0.84 | | 0.494 | 0.50 | | 1.40 | |  |
|  | |  |  | |  | |  |  |
| Severe plasma leakage | Odds Ratio | | P | 95% CI | | | |  |
| Age | 1.00 | | 0.789 | 0.99 | | 1.01 | |  |
| Male | 0.60 | | 0.000 | 0.46 | | 0.77 | |  |
| Migrant | 0.66 | | 0.009 | 0.48 | | 0.90 | |  |
| Diabetes mellitus | 1.17 | | 0.624 | 0.63 | | 2.15 | |  |
| Hypertension | 1.30 | | 0.221 | 0.85 | | 1.99 | |  |
| Charlson’s comorbidity Score | 1.31 | | 0.003 | 1.10 | | 1.57 | |  |
|  | |  |  | |  | |  |  |
| ICU Admission | | Odds Ratio | P | 95% CI | | | |  |
| Age | | 1.04 | 0.087 | 0.99 | | 1.10 | |  |
| Male | | 1.16 | 0.828 | 0.31 | | 4.32 | |  |
| Migrant | | 0.92 | 0.919 | 0.17 | | 4.91 | |  |
| Diabetes mellitus | | 2.28 | 0.406 | 0.33 | | 16.02 | |  |
| Hypertension | | 1.15 | 0.881 | 0.19 | | 6.79 | |  |
| Charlson’s comorbidity Score | | 1.24 | 0.465 | 0.69 | | 2.24 | |  |
|  | |  |  | |  | |  |  |
| LOS hospitalization | β coefficient | | P | 95% CI | | | |  |
| Age | 0.02 | | 0.000 | 0.01 | | 0.02 | |  |
| Male | 0.11 | | 0.195 | -0.06 | | 0.28 | |  |
| Migrant | 0.27 | | 0.003 | 0.09 | | 0.44 | |  |
| Diabetes mellitus | 0.66 | | 0.013 | 0.14 | | 1.17 | |  |
| Hypertension | -0.08 | | 0.623 | -0.39 | | 0.23 | |  |
| Charlson’s comorbidity score | 0.80 | | 0.000 | 0.62 | | 0.98 | |  |

Linear regression for continuous variable and logistic regression for categorical variables. Adjusted to patient’s background variables, i.e. age, gender, migrant status and co-morbidities.

DHF: dengue hemorrhagic fever; DSS: dengue shock syndrome; ICU: intensive care unit; LOS: length of stay.
